# Prenatal Iron Deficiency in Guinea Pigs Increases Locomotor Activity but Does Not Influence Learning and Memory

**DOI:** 10.1371/journal.pone.0133168

**Published:** 2015-07-17

**Authors:** Catherine Fiset, France M. Rioux, Marc E. Surette, Sylvain Fiset

**Affiliations:** 1 Programme de nutrition, Faculté des Sciences de la Santé, Université d’Ottawa, Ottawa, Ontario, Canada; 2 Département de Chimie et Biochimie, Université de Moncton, Moncton, New Brunswick, Canada; 3 Secteur des Sciences Humaines, Université de Moncton, Campus d’Edmundston, Edmundston, New Brunswick, Canada; Northeastern University, UNITED STATES

## Abstract

The objective of the current study was to determine whether prenatal iron deficiency induced during gestation in guinea pigs affected locomotor activity and learning and memory processes in the progeny. Dams were fed either iron-deficient anemic or iron-sufficient diets throughout gestation and lactation. After weaning, all pups were fed an iron-sufficient diet. On postnatal day 24 and 40, the pups’ locomotor activity was observed within an open-field test, and from postnatal day 25 to 40, their learning and memory processes were assessed within a Morris Water Maze. The behavioural and cognitive tests revealed that the iron deficient pup group had increased locomotor activity, but solely on postnatal day 40, and that there were no group differences in the Morris Water Maze. In the general discussion, we propose that prenatal iron deficiency induces an increase in nervousness due to anxiety in the progeny, which, in the current study, resulted in an increase of locomotor activity.

## Introduction

Affecting two billion people—or over 30% of the world’s population—iron deficiency (ID) anemia (IDA) is the most prevalent nutrient disorder in the world [1]. Distributed throughout both developing and industrialized countries, it is especially common among pregnant women and children. It is estimated that 52% of pregnant women in developing countries and 23% in industrialized countries suffer from IDA [1]. These alarming statistics raise concerns pertaining to the effects of maternal and childhood IDA on child development.

In humans, brain development occurs primarily prenatally and during early childhood. Iron is a vital nutrient in this process. In the foetal phase, iron is responsible for tissue oxygenation, energy metabolism, neurotransmitter synthesis, and myelination of the central nervous system [2, 3]. For pregnant women, this means that their iron requirements increase dramatically in order to provide for fetal brain development [4]. In fact, pregnant women must consume three times their regular iron requirements, which is a major contributing factor to world-wide IDA [1]. Children who were iron deficient in the womb remain iron deficient throughout the first year of life despite iron supplementation [5, 6]. This is a time when brain development is still in its prime: from birth until 24 months of age, rapid brain growth occurs in infants, resulting in the development of significant cognitive and motor skills. This is also when brain iron concentrations peak, indicating a relationship between iron and the neurophysiological processes of behavioural organization [7]. Conversely, ID during infancy has been proven to significantly reduce iron concentrations in the cerebral cortex and hippocampus, two brain regions associated with learning and memory [8].

The effects of IDA in human children have been extensively reviewed, but few explore the effects of prenatal IDA, particularly from a cognitive perspective. Several studies on prenatal ID have seen no effect [9], but irritability [10], immature reflexes [10, 11], and poor performance on psychomotor developmental scales [9] and cognitive tests [12, 13] have been observed in infants. Infants born to iron deficient mothers have also been shown to express decreased alertness and soothability [14]. Due to cofactors such as socioeconomic status and uncontrolled degrees of ID, the impact of prenatal ID in children remains inconclusive [3, 7, 9, 10, 12, 15, 16]. Several studies have, however, demonstrated a relationship between low maternal iron status and low birth weights in infants [5, 6, 17].

Knowledge pertaining to ID postpartum in human children is more widespread. It is associated with poor performance in developmental tests [18, 19], learning tasks [18, 19], and long-term educational achievement [19, 20]. Additionally, children with postnatal ID display delayed maturation in auditory brainstem response tests (ABR) [21]. Unfortunately, conclusions on the impact of prenatal (as well as postnatal) IDA in humans are based on correlational studies, reinforcing the need for experimental research with animals [16].

Consequently, studies have been conducted using animal models to mimic the effects of IDA on development. Previously, many animal studies opted to focus on postnatal IDA. As with children, these studies have demonstrated that postnatally iron deficient animals had lower weights than those fed iron sufficient diets [22, 23]. Postnatal ID research has also exhibited learning deficits in T-tests with piglets [24], reduced swimming speed and impaired memory and learning capabilities in water maze problems in rats [25–27], increasing inactivity in rats [22, 28], and weakened grip-strength in mice [29]. Recently however, the amount of studies observing the effects of postnatal ID has significantly decreased, and the focus has shifted towards the effects of prenatal ID [30]—a prevailing worldwide issue [1].

The peak of brain development in several animal models, for example the rhesus monkey [31, 32], piglet [24], and guinea pig [33–37], occurs during the gestational period, similarly to that of humans, making them strong models for prenatal ID studies. In past studies with rhesus monkeys and our previous studies with guinea pigs, the effects of prenatal ID on cognition and behaviour were analyzed. Both the rhesus monkey and guinea pig had no visible cognitive effects. However, they had opposing results concerning behaviour: the rhesus monkeys’ activity levels decreased [32] whereas the guinea pigs’ remained the same shortly after birth [37] and increased at an older age [36]. Newborn guinea pigs born to dams fed an iron deficient diet also behaved similarly to their iron-sufficient counterparts in object recognition and novelty tasks [37], but prenatally iron-deprived rhesus monkeys were found to be less fearful and tended to manipulate novel objects more frequently [31].

Surprisingly, rats and mice, whose brain development occurs mainly after birth, especially when compared with rhesus monkeys, piglets, and guinea pigs, demonstrate ample effects in prenatal ID studies. Rats presented poor water-maze [38, 39], trace conditioning [40], and passive avoidance abilities [41], and displayed pre-pulse inhibition (PPI) deficits in acoustic-startle response tasks [42]. They were also hesitant in novel settings [38], increasingly inactive and anxious [43], had low birth weights [30, 44], poor sensorimotor development scores [38, 39], and had high mortality rates [30]. Prenatally iron deficient mice also had low water-maze performance scores, had attenuated startle responsiveness, and demonstrated reduced grip-strength [23].

The results of the studies presented above pertaining to the effects of prenatal ID are in discordance with those of our previous study with 24–40 day-old guinea pig pups [36]. Most studies have demonstrated a decrease in learning abilities, whereas we had no effects. With regard to behaviour, most studies suggest a diminution in activity, but our pups exhibited elevated activity levels. However, our previous studies with newborn guinea pigs [37] demonstrated no effect on activity. Mobility was also reduced in many studies—yet again our results were contradictory: the guinea pigs presented no visible effects. Our previous study [36] had surprising results; however, data was collected manually, making it susceptible to human error [45]. Leblanc et al. [36] was limited to timing how long it took guinea pigs to reach the platform in the Morris Water Maze (MWM) and manually counting the number of cell crossings in the Open Field Test (OFT).

The general purpose of the present study was to reassess the impact of prenatal and early postnatal iron deficiency anemia in the domestic guinea pig with regards to locomotor activity and learning and memory processes. To reach this objective, we replicated the study by Leblanc et al. [36], but refined the methodology: we used more precise measuring tools, which allowed us to analyze more parameters (MWM: swimming speed, floating time, length of the swimming path, the percentage of time spent in each zone and quadrant, heading; OFT: percentage of time in movement, distance covered, and percentage of time spent in the guinea pig’s most-visited cell) than in Leblanc et al. [36]. The addition of dependent variables allowed us to further identify which aspects were affected by maternal IDA, which was not possible in the former study. As outlined by Walsh and Cummins [46] and Stover et al. [47], the addition of dependant variables yields more information about the results.

Of particular interest was the addition of the dependent variable, “path distance” in the MWM in the current study. Swim path distance and escape latency are the most common indexes of cognitive performance in the MWM [48]. In Leblanc et al. [36], escape latency was used as a measure of spatial memory learning. However, this is not necessarily a good measure of learning ability [49]. As described by Lindner [50–52], the length of the swimming path taken by the animal is a better determinant of learning ability. In contrast with Leblanc et al. [36], the current study analyzed the length of the swimming path in addition to escape latency (in order to compare with the previous study).

Additionally, as stated by Shadish et al. [53], the change of instrumentation in a replication study may alter the outcome, justifying the current study and its use of automatic tracking tools instead of manual scoring as in Leblanc et al. [36]. Several studies outline the advantages of automatic tracking systems over manual methods, stating that they are more accurate [45], consistent [45, 54] and identify behavioural information that may otherwise be missed [45, 54, 55]. Since we improved the precision of our instrumentation and added dependent variables by passing from manual scoring to automatic detection, the current study allowed us to further identify which aspects of locomotor activity and learning and memory processes are affected by prenatal iron deficiency anemia in guinea pig pups.

## Materials and Methods

### Ethical statement

This study was approved by the Comité de protection des animaux (CPA) of the Université de Moncton (Permit Number: 07–02), which is responsible for the application and enforcement of the rules of the Canadian Council on Animal Care. All animals were given *ad libitum* access to water and food. They were caged in groups of two animals or more and had access to a running wheel. Before surgery, animals were anesthetized with a solution of 10:1 ketamine/xylazine. They were sacrificed by decapitation. All efforts were made to minimize suffering.

### Animals

Twenty-four female and two male Hartley guinea pigs (*Cavia porcellus*, 10 weeks) were purchased from Charles River Laboratories (St Constant, QC, Canada). Due to complications in the mating phase, four additional males of the same age were purchased (see Mating and Delivery). All animals were housed in a controlled environment (relative temperature and humidity of 22°C and 50%, respectively, with a 12-hour light cycle with lights on at 0700) at the animal-care facility at the Université de Moncton, Edmundston Campus, for the duration of this study.

### Diets

Guinea pigs were fed either iron-sufficient (IS) or iron-deficient anemic (IDA) diets purchased from Harlan Teklad (Madison, WI, USA), which were consumed *ad libitum* along with fresh water. The iron contents of the IS and IDA diets were 130.0 and 10.1 mg iron/kg feed, respectively. The iron level for the IDA diet was adapted from MacDonald et al. [56] in order to induce a light-to-moderate iron deficiency in guinea pigs. The IS diet was also adapted from MacDonald et al. [56], and in accordance with the required iron intake for guinea pigs described in The Nutrient Requirements of Laboratory Animals [57]. The nutritional composition of both diets was identical to that previously reported by Leblanc et al. [36]. Food intake was recorded daily and body weight every third day.

### Procedure

#### Mating and Delivery

The mating and delivery period was divided into 4 phases: the habituation, mating, gestation, and lactation phases.

The day after their arrival to the university’s animal care facility, female guinea pigs were randomly and equally divided into two diet groups: IS and IDA. They were then separated into groups of three and fed their respective diets for 3–5 days until their food intakes stabilized (the habituation phase). Males were kept in individual cages away from the females during this phase and fed IS diets.

Following the habituation phase, the females were placed into large mating cages according to their diet groups, forming two cages of six. Each diet group of females was introduced to one male for mating, as suggested by the Canadian Council on Animal Care [58], for a duration of 35 days. During this phase, males were removed from their mating cages and fed IS diets for two hours every day to ensure that they did not develop an iron deficiency. Three females from the IDA group were removed seven days into the mating phase to conform to guidelines by the Canadian Council on Animal Care (endpoints) due to an average weight loss of 20.0%.

To adhere to a recommendation by the Canadian Council on Animal Care [58] suggesting that guinea pigs require social companions, females were placed into cages of two after the mating phase, where they continued to be fed their assigned diet throughout the gestation (63 days) and lactation (9 days) phases. During the lactation period, as described by previous studies [59–61], pups began eating their mother’s assigned diet as well as her milk, meaning that prenatally IDA pups also consumed an IDA diet early in life. Although the intake could not be measured, the quantities were believed to be minimal. Pups were weaned at ten days postpartum and fed an IS diet for the remainder of the study (the testing period). Females were then sacrificed. Pups of similar age were caged together for the remainder of the study, forming cages of two to six in order to provide a social environment [58], and always had access to a running wheel for exercise.

To allow experimenters ample time to assess all subjects in the behavioural tests, the sample was divided into two, meaning that half the females began the study one month after the other half. Both groups of animals, however, were the same age at the beginning of the study. Originally, in order to avoid genetic confounding factors, males were to be returned to their cages after the first mating phase, fed an IS diet, and then crossed to the other diet group for a second mating phase. Unfortunately, in the period between the beginning and end of the first mating phase, both males lost a significant percentage of weight (M = 21.7%), and from fear for their health and in order to conform to guidelines set by the Canadian Council on Animal Care (endpoints), four new male guinea pigs were purchased. Two of these replaced the weakened males midway through the first mating phase (day 13 of the mating phase), and the other two were coupled with the second group of females during the second mating phase.

As in the first mating phase, the new males were randomly assigned to female groups. In addition, the mating period was extended to sixty days for the first mating group in order to allow the new males to mate with the females. The extension of the mating phase meant that the average length of time that females were on their assigned diets prior to the gestational period varied between mating groups, but for the IS group only. A Mann-Whitney test for independent variables (since the data were not evenly distributed) revealed that the values were not significantly different between groups (*U* = 24, *p* = 0.16). Additionally, an unpaired t-test revealed that the mean age of females at the beginning of pregnancy was the same for both groups (ID: M = 102.50 days, IS: M = 108.80 days), *t*(16) = 0.984, *p* = 0.340, 95% CI of the mean difference = -7.27, 19.87.

#### Testing

During the testing period, the guinea pig pups’ behaviour and learning abilities were assessed by means of Open Field Tests and Morris Water Maze tests. Testing began on postnatal day 24 (PNd24) and ended on PNd40. This timeline was chosen to ensure that pups had the physical strength to swim in the pool (H.C. Dringenberg, personal communication, May 20, 2006). Moreover, PNd24 also allowed our pups to be on a solid food diet for at least two weeks prior to testing, and for the end of testing to fall shy of the beginning of adolescence in the guinea pig (PNd40) [62, 63]. All our tests were thus conducted during the guinea pigs’ childhood.

#### Locomotor activity

The pups’ spontaneous locomotor activity was assessed via an Open Field Test (OFT), a common method of measurement for the exploratory behaviour and general activity of rodents [64]. Although the OFT is typically used to assess locomotor activity in rats and mice, previous studies have adapted it for use with guinea pigs [37, 63, 65, 66]. By observing their movement in an open enclosure, locomotor activity and emotionality can be measured [46, 63, 64]. Animals that spend the majority of their time in the periphery of the enclosure are more anxious than those which explore the centre [64].

The open field utilized for this study consisted of a 20 cm high, 1 m x 1 m enclosure made of painted-black plexiglass. The bottom of the field was covered in 3 cm of pine wood chips and divided into a 5 x 5 grid. Between the testing of each animal, the enclosure was cleaned and wood chips were replaced. On PNd24 and PNd40 (four hours after the end of the water maze test, see below), pups were permitted to roam the enclosure for one session of 15 minutes, which was recorded from above and analyzed using a computer-based tracking system (HVS Image, Hampton, UK). The dependent variables were: the distance covered (m), the percentage of time in movement, the total number of cell crossings, the percentage of cells visited, and the percentage of time spent in the four corners of the enclosure, in the peripheral and central cells, and in the guinea pig’s most-visited cell. In order for the tracking system to accurately detect movement, OFTs took place in a dimly-lit room with four lamps pointed towards the ceiling.

#### Morris Water Maze

The Morris Water Maze (MWM), initially developed by Morris [67] to assess rodents’ spatial learning, was used to test the guinea pig pups. By relying on distal cues, rodents in the MWM must navigate from various start locations on the perimeter of an open swimming arena to locate a submerged escape platform [68]. By analyzing the animal’s behaviour, deductions can be made over it’s ability to learn spatially. The MWM was first adapted for guinea pigs by Dringenberg, Richardson, Brien and Reynolds [69], and the same procedure was followed in the current study.

A black circular pool (165 cm diameter, 75 cm deep) was filled with water (32 cm deep, temperature 23–25°C) and served as the swimming arena. A circular escape platform (15 cm diameter) made of plexiglass was submerged 2 cm below water level and located in the centre of one of the four pool quadrants (numbered in a clockwise direction). The offsprings’ swim path was recorded and analyzed by means of a second computer-based video tracking system (HVS Image, Hampton, UK). The dependent variables were: the length of the swim path (m), latency to reach the platform (sec.), the percentage of time spent floating, the average swimming speed excluding floating time (active swim, m/sec.), and the percentage of time the animal spent in each quadrant. The percentage of time spent in each zone (labelled A, B, and C, where zone C was the outer ring and zone A the interior ring) as well as heading (deg.) was also measured.

In the current study, testing in the Morris Water Maze consisted of three phases: the acquisition phase, the retention phase, and the inversion phase. Pups began the first phase of testing, the acquisition phase, on PNd25. For ten consecutive days, guinea pigs received one block of four training trials wherein the escape platform’s location remained the same and the guinea pigs’ ability to learn to locate the escape platform was evaluated. The platform’s position in the four pool quadrants was counterbalanced between guinea pigs. In each trial, guinea pigs began facing the exterior wall of the swimming pool and then were allowed to swim freely for 45 seconds. If the guinea pig found the platform, it was left there for 15 seconds and then removed and dried under a heat lamp until the next trial. If the guinea pig did not find the platform after the allotted 45 seconds, it was placed on it for 15 seconds and then removed and dried for the following trial.

On PNd39, five days after the last training trial, pups underwent the retention phase. This consisted of four 60-second trials in which the platform was removed from the pool. The retention phase was done to assess reference memory [68]. By observing in which quadrant the guinea pigs swam, it could be seen if they acquired the ability to use distal cues over time to locate the platform. At the end of each trial, pups were also dried under a heat lamp as in the acquisition phase.

On PNd39 (immediately after the end of the retention phase) and PNd40, the guinea pigs participated in the inversion phase. The escape platform during this trial was placed in the opposite quadrant as in the training phase, and guinea pigs were tested for two sessions of four 45-second trials to see if they could override their existing knowledge about the previous platform’s location and learn the placement of the new one. The same procedure was used in the inversion phase as in the acquisition phase.

### Blood collection

Pups, at the age of 41 days, and females, 10 days after delivery, were anesthetized with a solution of 10:1 ketamine/xylazine (1 ml/kg body weight) and sacrificed by decapitation. A 1-ml blood sample was then collected in an EDTA-anticoagulated tube and sent to the Edmundston Regional Hospital in Edmundston, New Brunswick, Canada, for complete blood count (hemoglobin (Hb), hematocrit (Hct), and mean cell volume (MCV)) using a Beckman-Coulter Ac-T diff2 hematology Analyzer. Spectrophotometry was used to analyze Hb and aperture independence was used for MCV and red blood cells (RBC). Hct was calculated by multiplying MCV by RBC and dividing by 1000.

### Statistical analysis

In contrast with our recent publications [34, 35, 70] in which, to avoid genetic confounding factors, sibling data were combined as a single unit, the current study does not use this approach. Instead, data from individual guinea-pigs were used because learning and behavioural processes are specific to individuals and cannot be combined across multiple animals.

SPSS (v. 21.0, Chicago, IL, USA) and Prism (v. 6.0e, La Jolla, CA, USA) were used to analyze the data. For all statistical tests, an alpha level of 0.05 was adopted to reject the null hypothesis. In all mixed ANOVAs, when the matrix of variance-covariance was heterogeneous, the degrees of freedom of the within-subject variable were adjusted using the Greenhouse-Geisser index. Moreover, significant interactions were followed by a series of post hoc t-tests corrected with the Bonferroni method [71]. When possible, the sex of the pups was included as an independent variable in the statistical analyses, but later dropped from the statistical model when there were no significant sex effects or interactions with sex. Finally, in both IS and IDA groups, there were no significant differences between guinea pigs genetically related to different males. Consequently, it appears that the possible genetic confounding factor due to the males did not influence the performance of the guinea pigs in the current experiment. This variable was also dropped from the statistical model.

## Results

### Maternal Weight

Dams in both groups weighed the same (IS: M = 594.63 *g*, SEM = 13.87; ID: M = 601.85 *g*, SEM = 12.40) during the habituation phase, *F*(1, 16) < 1, *p* = 0.70. Moreover, the dams’ weight gain between the first (M = 590.66 *g*, SEM = 10.07) and last day (M = 605.81 *g*, SEM = 13.46) of the habituation phase was not large enough to be significant, *F* (1, 16) = 1.05, *p* = 0.32, and there was no group x day interaction, *F* = (1, 16) < 1, *p* = 0.78.

As expected, both groups of dams gained a significant amount of weight between the first day of gestation (M = 673.56 *g*, SEM = 21.27) and the day before delivery (M = 1156.90 *g*, SEM = 31.81), *F*(1, 16) = 524.07, *p* < 0.001. However, there was no significant difference between the two diet groups in terms of mean weight gain during the gestation phase (ID: M = 880.31 *g*, SEM = 37.14; IS: M = 950.15 *g*, SEM = 33.22), *F*(1, 16) = 1.96, *p* = 0.18, or group x day interaction, *F*(1, 16) = 1.51, *p* = 0.28. In both IS and IDA groups, the weight gain during gestation was similar, suggesting that the IDA diet did not influence weight gain.

### Dam Haematological Data

The dams’ haematological data were analyzed on PNd10. Since there was a strong correlation between Hct and Hb for both IS (*r* = 0.985, *p* < 0.001) and IDA groups (*r* = 0.942, *p* < 0.001), a MANOVA was performed on both groups with Hct and Hb as composite variable. There was a significant diet group effect, Wilk’s lambda = 0.19, *F*(2, 15) = 31.94, *p* < 0.001. Subsequent univariate ANOVAs revealed lower Hct (*F*(1, 16) = 41.44, *p* < 0.001) and Hb levels (*F*(1, 16) = 56.43, *p* < 0.001) in the IDA dams than in the IS dams (Table 1). The IDA dams’ mean Hb levels were well below the normal range, which normally vary between 110 and 140 *g*/L, with a mean of 123 *g*/L in guinea pigs [56]. IS dams had normal Hb levels. These results clearly indicate that the IDA group were substantially iron deficient.

**Table 1:** Iron status for IS and IDA guinea-pig dams^1^ and pups^2^ (M).

	**IS**	**IDA**	***P*-value**
**Dams**			
Hb (*g*/L)	134.20	93.25	< 0.001
Hct	0.389	0.286	< 0.001
**Pups**			
Hb (*g*/L)	125.62	122.93	n.s.
Hct	0.370	0.360	n.s.

^1^ IS (*n* = 10) and IDA (*n* = 8)

^2^ IS (*n* = 26) and IDA (*n* = 14).

### Offspring Outcomes

Litter size (*t*(16) = 0.43, *p* = 0.63, 95% CI = -0.58, 0.93), litter birth weight (*t*(16) = 0.17, *p* = 0.87, 95% CI = -111.43, 94.64), pre- and postnatal mortality (Fisher exact probability test, *p* = 0.19), and gender distribution (*X* ^2^(1) = 0.80, *p* = 0.37) did not differ between the groups (Table 2). There was, however, a significant difference between the total mortality rates and number of pups alive on PNd40 between the two diet groups (Fisher exact probability test, *p* = 0.003). Pups with mothers in the IDA group had higher rates of mortality in comparison with those from the IS group. More specifically, in the IDA group, one pup died at birth, five died before PNd13 and three died between the third and the sixth MWM test. In the IS group, only one pup died at birth. All deaths were from unknown reasons. Consequently, all statistical analyses on pups were performed with 26 and 14 pups born to IS and IDA dams, respectively.

**Table 2:** Birth outcomes for IS and IDA guinea-pig dams^1^ (M ± SEM).

	**IS**	**IDA**	***P*-value**
**Litter size (pups/litter), n**	2.7 ± 0.3	2.9 ± 0.2	n.s.
**Pups alive at birth (pups/litter), n**	2.6 ± 0.2	2.8 ± 0.2	n.s.
**Mortality/survival distribution, n**			
Prenatal Mortality	1	1	n.s.
Postnatal Mortality	0	8	
Before PNd24	0	5	n.s.
After PNd24	0	3	
Total Mortality	1	9	0.003
Number of pups alive on PNd40	26	14	
**Gender distribution on PNd40, n**			
Males	11	8	n.s.
Females	15	6	
**Litter birth weight (pups alive), g**	287.9 ± 78.1	287.8 ± 77.9	n.s.

^1^ IS (*n* = 10) and IDA (*n* = 8).

### Pup Weight

The weight of the pups was analyzed as a function of sex and group at birth (PNd1), at the beginning of the behavioural tests (PNd24) and at the end of the study (PNd40). An ANOVA group (IS, IDA) x sex (M, F) x day (PNd1, PNd24, PNd40) revealed a significant main effect of group, *F*(1, 36) = 7.01, *p* = 0.012, sex, *F*(1,36) = 4.56, *p* = 0.040, and day, *F*(2, 72) = 2322.88, *p* < 0.001. In addition, there was a significant group x day interaction, *F*(2, 72) = 3.34, *p* = 0.041, and a sex x day interaction, *F*(2, 72) = 6.62, *p* = 0.002. In summary, males were heavier than females, but solely on PNd40, and pups from both sexes in the IDA group weighed less than those from the IS group on PNd1, but weighed the same on PNd24 and PNd40. As expected, both groups gradually gained weight from PNd1 to PNd40. When data concerning the pups’ weights were observed in relation to their food intakes (see S1 Results), it became apparent that pups from the IDA group were smaller at birth, but ate more for the duration of the study, resulting in equal weights between both groups on PNd40. This assumption is supported by the fact that both groups had similar relative food intakes (total food intake / weight gain) from PNd10 to PNd40 (IS: M = 2720 *g*; IDA: M = 2689 *g*), *t*(17.5) = 0.112, *p* = 0.912, 95% CI of the difference = -0.38, 0.43). Thus, by PNd40, both groups were similar in terms of total food intakes relative to their weights. However, as stated in the S1 Results section, one should be very careful before drawing any serious conclusion from these data.

### Pup Haematological Data

The pups’ haematological data were analyzed on PNd41. As with the dams, there was a strong correlation between Hct and Hb for pups from both IS (*r* = 0.985, *p* < 0.001) and IDA groups (*r* = 0.942, *p* < 0.001). Therefore, a MANOVA with Hct and Hb as composite variable was performed, but indicated no significant difference between the two groups, Wilk’s lambda = 0.96, F(1, 37) < 1, *p* = 0.47. At the end of the study, pups from both groups had comparable Hct and Hb levels (Table 1), which were well within the normal range for guinea pigs [56].

Although we did not measure the iron status of the newborn guinea pigs, one of our previous studies with guinea pigs [37] also used the same IDA diet as the current study, and it clearly revealed that the pups born to IDA dams were IDA at nine days of age. In this study [37], the prenatally ID pups had significantly lower hemoglobin and hematocrit levels than IS pups (ID: M = 78.6 *g*/L, M = 0.24%; IS: M = 129.2 *g*/L, M = 0.37%). It is also documented that maternal iron status is directly related to newborn iron status [6, 30], and our females were significantly IDA during pregnancy. We are therefore quite confident that our IDA guinea pig pups were IDA at birth.

### Exploratory Behaviour in the OFT

Analyses of pups’ behaviour in the OFT did not follow the conventional approach. Firstly, although Gould, Dao and Kovacsics [64] suggested that exploratory behaviour in OFTs be analyzed over time using 5-minute intervals, we did not. The reason behind this is that our animals made very few movements during both 15-minute sessions. This is typical of guinea pigs, whose regular behavioural patterns are to explore little and freeze in novel settings [60, 65]. Consequently, the dependant measures in the OFT were analyzed for a total of 15 minutes during each session. Secondly, although there were no outliers (using three standard deviations from the mean as criterion), a series of Kolmogorov-Smirnov tests revealed that the data for most dependent variables from both groups were far from being normally distributed (*p*_s_ range: 0.042 to < 0.001) during both OFT sessions. Even though we applied all data transformations proposed by Daumas [72], we were not able to normalize the data distribution and meet the assumption of normality in order to perform a mixed ANOVA. Consequently, data for all dependant measures from both groups on PNd24 and PNd40 were independently compared via a series of nonparametric tests (Mann-Whitney). To reduce the chance of making a Type 1 error, we applied the Bonferroni correction (alpha set at 0.025) for all comparisons.

On PNd24, there was no significant difference between the two groups on the distance covered (*U* = 154, *p* = 0.439), the percentage of time in movement (*U* = 151, *p* = 0.391), the percentage of cells visited (*U* = 155, *p* = 0.447), the total number of cell crossings (*U* = 150, *p* = 0.373), or the percentage of time spent in the peripheral (*U* = 167, *p* = 0.678) and central cells (*U* = 169.5, *p* = 0.730). Both groups were also equivalent in terms of the percentage of time spent in the four corners of the enclosure (*U* = 181, *p* = 0.984) and the percentage of time spent in the guinea pig’s most visited cell (*U* = 176, *p* = 0.865). Thus, it can be concluded from these analyses that, on PNd24, both IS and IDA groups performed similarly in the OFT.

**Fig 1:**
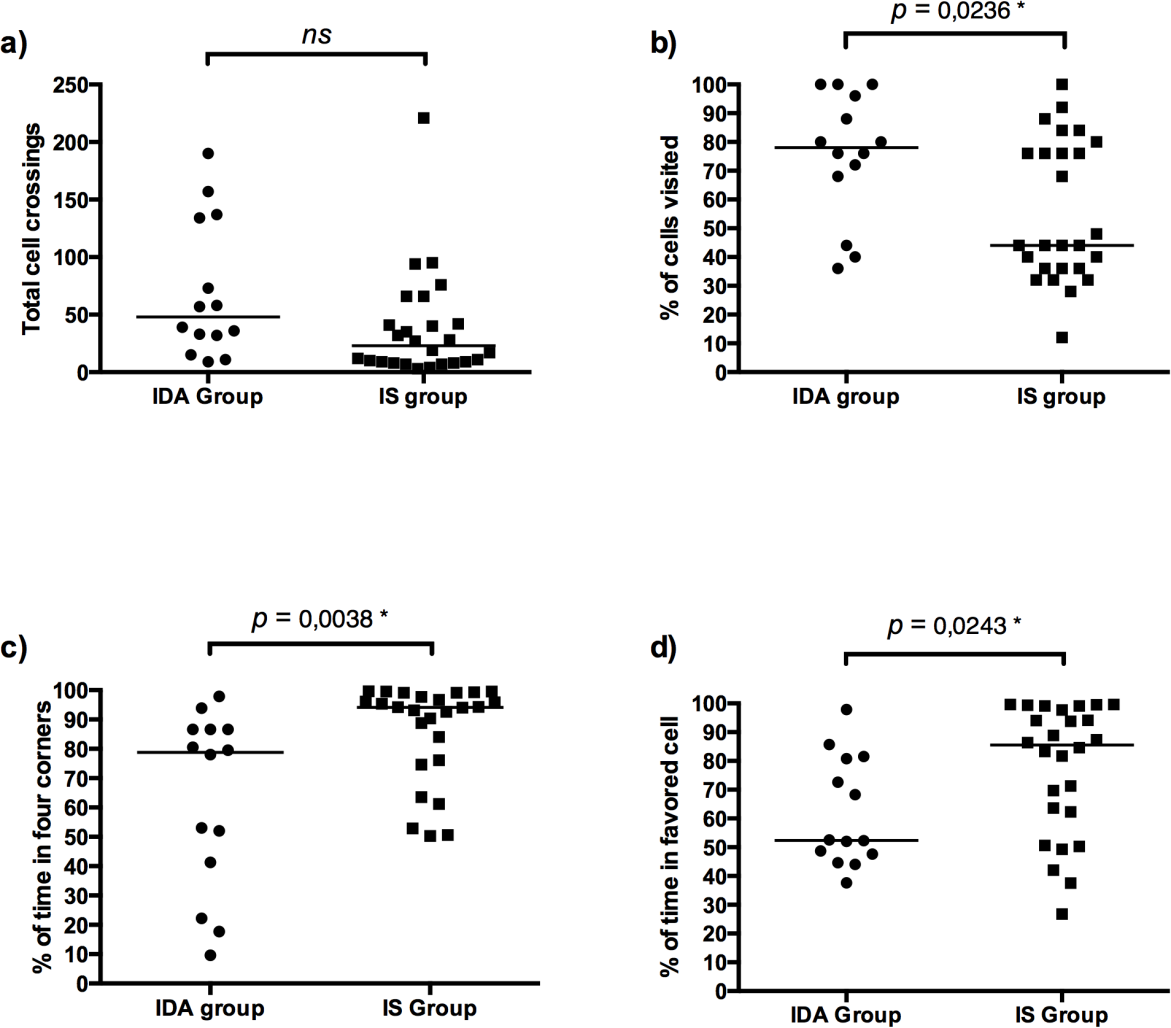
Median (horizontal bar) and individual scores of all IS (black square dots) and IDA pups (black circle dots) for (a) the total cell crossings, (b) the percentage of cells in the enclosure visited, (c) the percentage of time in the four corners of the enclosure and (d) the percentage of time spent in the guinea pig’s most visited cell in Session 2 of the OFT (PNd40). * Significant after Bonferroni correction for non-independent tests (*α* = 0.025).

On PNd40, however, the groups differed significantly on a few variables in the OFTs (see Fig 1). Although the total number of cell crossings did not differ among the two groups (*U* = 109, *p* = 0.038), pups in the IDA group visited a higher percentage of cells in the enclosure (*U* = 103, *p* = 0.024) than those in the IS group. The percentage of time in movement (*U* = 136.5, *p* = 0.20), as well as the distance covered by the pups (*U* = 119, *p* = 0.076), did not differ among the groups. On the other hand, pups in the IS group spent more time in the four corners of the enclosure (*U* = 82, *p* = 0.004), particularly in a single cell (*U* = 103, *p* = 0.024). The percentage of time spent in the peripheral (*U* = 120, *p* = 0.072) and central cells (*U* = 131, *p* = 0.140), however, did not differ between the groups. Although both groups spent the majority of their time within peripheral cells (IS, M = 98.23%, SE = 0.53; IDA, M = 99.36%, SE = 0.30), a behaviour associated with high anxiety levels [64], these analyses revealed that pups from the IDA group explored the enclosure more on PNd40 than those from the IS group, which had a tendency to remain in one corner of the enclosure.

### MWM

Though several dependant variables were measured during this phase of testing, solely two of them are presented here: the path distance (in meters)—which is recognized as a strong indicator of spatial memory in the MWM [50–52]—and escape latency (in seconds)—which allowed us to compare our results with those observed by Leblanc et al. [36].

When analyzing data in the MWM, it became clear that the data from several guinea pigs had to be removed. First, three siblings (2 males and 1 female) from the IS group were also withdrawn from all MWM analyses. Instead of actively searching for the platform, these pups spent over 50% of the time in each testing session floating (swimming speed was below 0.05 metres per second), exceeding 85% on several testing sessions. Also, as a reminder, three pups (1 male and 2 females) from the IDA group died during the testing phase, so their data were removed from the data set. Thus, the statistical analyses for the data of the MWM were run on 23 pups from the IS group and 14 pups from the IDA group.

In order to detect any differences between the two groups before beginning the MWM task [see 68], the path distance and escape latency in the first two trials (grouped together) of the first session was first examined. An independent sample t-test performed on the path distance revealed that both groups swam the same distance (*t*(35) = 1.260, *p* = 0.216). Regarding escape latency, however, since the data were not normally distributed (Kolmogorov-Smirnov, IS group, *K*(23) = 0.380, *p* < 0.001; IDA group, *K*(14) = 0.482, *p* < 0.001), a nonparamatric test (Mann-Whitney) was used. Both groups (medians = 45 sec.) performed similarly in terms of escape latency (*U* = 138, *p* = 0.397). Thus, the maternal IDA condition did not influence the IDA group’s performance at the beginning of the MWM. Consequently, any potential differences observed between the two groups during the MWM could not be attributed to the fact that the two groups already differed on Day 1 of this phase.

**Fig 2:**
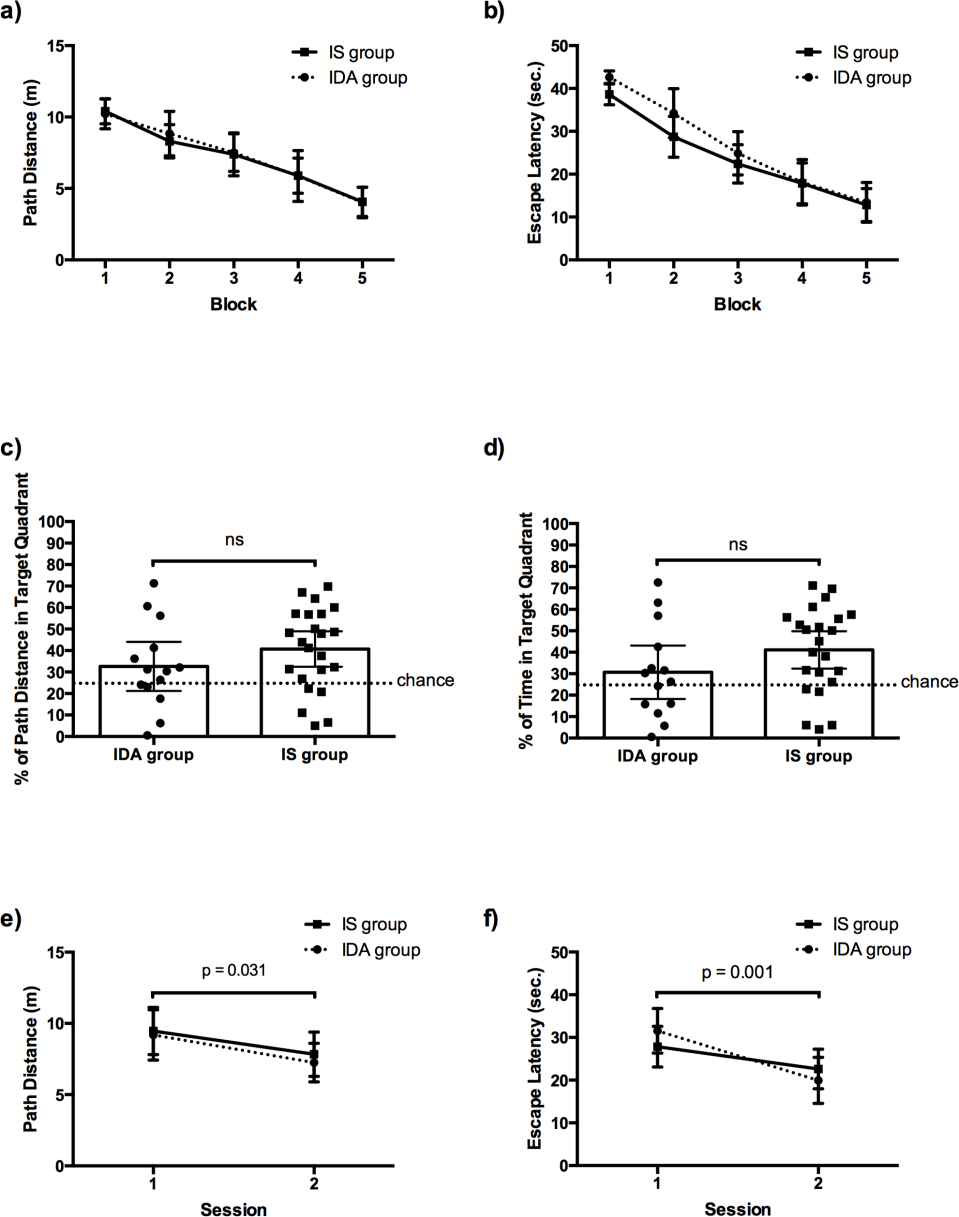
Mean path distance (a) and mean escape latency (b) of IS and IDA groups as a function of blocks of trials in the acquisition phase of the MWM. Mean percentage of path distance in target quadrant (c) and mean percentage of time spent in the target quadrant (d) as a function of group during the MWM retention phase (Dots illustrate individual scores of all IS (black square dots) and IDA pups (black circle dots)). Mean path distance (e) and mean escape latency (f) of IS and IDA groups as a function of sessions in the inversion phase of the MWM. Error bars represent 95% confidence intervals.

The data from the acquisition phase were then grouped into blocks of two days for a total of 5 blocks and were analyzed with a mixed ANOVA Group (IS, IDA) x Block (1 to 5) with repeated measures on the last factor. In regards to the path distance (Fig 2A), there was no difference between the groups (*F*(1, 35) < 1, *p* = 0.895), and no interaction (*F*(4, 140) = 0.149, *p* = 0.963). However, the Omnibus test for block was significant (*F*(4, 140) = 41.92, *p* < 0.001) and a series of Bonferroni tests showed that the mean path distance decreased from block 1 to block 5. The same pattern of results was observed for escape latency: There was a significant effect of block (*F*(4, 140) = 99.62, *p* < 0.001, but no effect of group (*F*(1, 35) = 1.167, *p* = 0.287, or interaction effect (*F*(4, 140) = 1.048, *p* = 0.3846. A series of Bonferroni tests revealed that escape latency gradually declined from block 1 to block 5 during the acquisition phase (Fig 2B).

In the retention phase, although a series of one sample t-tests revealed that the percentage of path distance in the target quadrant was at chance (set at 25%—one quadrant out of four) for the IDA group (*t*(13) = 1.447, *p* = 0.172, 95% CI -3.774 to 19.07) but over chance for the IS group (*t*(22) = 3.994, *p* < 0.001, 95% CI = 7.455 to 23.99), the two groups did not differ (*t*(35) = 1.229, *p* = 0.227, 95% CI 21.23 to 44.07) (Fig 2C). Similarly, the mean percentage of time in the target quadrant was at chance for the IDA group (*t*(13) = 0.986, *p* = 0.342, 95% CI 18.24 to 43.12) but over chance for the IS group (*t*(13) = 3.828, *p* < 0.001, 95% CI 32.38 to 49.82), but there was no difference between the two groups (*t*(35) = 1.485, *p* = 0.146, 95% CI -24.66 to 3.821) (Fig 2D). The fact that the IS pups searched above chance and the IDA pups did not may simply be attributed to the small effective sample size of our IDA pups for the one sample t-tests (path distance: *d* = 0.387; escape latency: *d* = 0.264). Thus, it appears that the impact of the maternal IDA was not significant enough to dissociate both groups’ capacity to remember the location of the platform five days after the last training trial.

During the inversion phase, an ANOVA group (2) x session (2) performed on the path distance (see Fig 2E) revealed that both groups learned to find the platform from session 1 to session 2 (*F*(1, 70) = 4.856, *p* = 0.031). However, there was no difference between the groups, *F*(1, 70) < 1, *p* = 0.591, nor interaction, *F*(1, 70) < 1, *p* = 0.847. Similar results were observed for the escape latency (Fig 2F): There was a significant effect of session (*F*(1, 70) = 11.62, *p* = 0.001) but there was no difference between the groups, *F*(1, 70) < 1, *p* = 0.834, nor interaction, *F*(1, 70) = 1.69, *p* = 0.198. Both groups learned to find the relocated platform in the pool at the same rate during the inversion phase.

Finally, within all of the phases, all other dependant variables of the MWM (the percentage of time spent floating, the average swimming speed (m/sec.), the heading (deg.), the percentage of time the animal spent in each quadrant and the percentage of time spent in each zone (labelled A, B, and C)) showed the same pattern of results—there were no differences between the two groups—as the ones illustrated above with path distance and escape latency (data not reported here).

## Discussion

The main objective of the current study was to identify the effects of prenatal and early postnatal IDA in guinea pig pups by replicating and modifying—by using automatized behavioural measurement tools and analyzing additional parameters—the study by Leblanc et al. [36]. Of interest was to determine if we would obtain the same surprising results, which suggested that prenatal IDA provokes an increase in exploratory behaviour in guinea pig pups but does not influence their learning or memory abilities. We partially replicated these results: our guinea pig pups born to IDA dams performed as well as those born to the IS dams within the MWM and displayed increased activity levels within OFTs. However, they were more active solely on PNd40, not PNd24 and PNd40 as in Leblanc et al. [36].

To explain why the prenatally IDA pups displayed an increase in exploratory behaviour in the OFTs, we propose that they could not suppress their nervous reactions to the novel environment, which suggests that they had elevated anxiety levels in comparison to the IS pups. Recent studies by Harvey and Boksa [41, 42] have found that prenatal ID in rats resulted in the absence of sensory motor inhibition: their pups presented pre-pulse inhibition (PPI) deficits in acoustic-startle response tasks, which suggests that prenatal ID animals present heightened anxiety. Likewise, Bourque et al. [43] and Unger et al. [39] observed higher thigmotaxis—movement due to a stimulus—in prenatal ID and IDA rats, respectively, in MWM tests, which is also associated with anxiety. This is, however, contradictory with findings by Golub et al. [31], which suggest that prenatally IDA rhesus monkeys are less fearful of novelty and have decreased anxiety. Nonetheless, since our guinea pig pups remained in the peripheral cells of the enclosure during the OFTs and that this behaviour is associated with anxiety [64], we rather hypothesize that prenatal IDA provokes an increase in anxiety. To further test this hypothesis, we suggest the use of acoustic-startle responses as means for anxiety measurements in guinea pigs.

Although both the current study and the one conducted by Leblanc et al. [36] have similar results concerning an increase in exploratory behaviour in prenatal IDA pups, our IDA pups were more active solely on PNd40, and not on PNd24. Similarly, our group also found no significant difference between groups in younger pups (PNd4) in the OFT tasks [37]. The reason why we were not able to replicate our previous results on PNd24 is unclear since both studies followed the same procedure. Notably, the OFTs within the current study were conducted in a dimly-lit room to accommodate for the use of the computer-based tracking system, which may have contributed to a calmer environment, but this does not explain the absence of a difference on PNd24. One could also argue that, since we tested the pups twice on PNd40, first in the MWM and then four hours later in the OFT, our pups were energized by the additional activity and moved more within the OFT. However, other studies have administered several transfer tests in one day [69, 73] and this would not explain the difference observed between the two dietary groups, since both underwent the same procedure.

The increase in exploratory behaviour observed on PNd40 highlights an interesting point: prenatal IDA influences behaviours long after birth and even when iron status has been normalized. By PNd40, all pups had a normal iron status, yet they displayed behavioural differences. It is possible that the effects of prenatal IDA manifested only at an older age, as was observed by Harvey and Boksa [41, 42] in prenatally ID rats. In recent studies, they demonstrated that while prenatally ID rat pups did not display memory deficits in passive avoidance tests, adult rats, which were prenatally ID, did. The increase in activity observed in older guinea pig pups in the current study reflects the idea that the effects of prenatal IDA may have long lasting detrimental effects that may persist later in life. Our laboratory is currently investigating the impact of prenatal IDA in adult guinea pigs by using auditory brainstem responses to see if they display an increase in neurophysiological deficits at an older age.

Testing within the MWM also had similar results as the previous study by Leblanc et al. [36]. Despite the use of more precise tools to analyze a large number of dependent variables, we did not identify any learning or memory deficits. As was presented earlier, it is possible that impairments as a result of prenatal ID appear only as adults, as was observed by Harvey and Boska in rats [41]. Older guinea pig pups may present deficits, since we only tested young pups. Recent studies [74] also suggest that animals that do not show evidence of cognitive impairment when tested in basic MWM problems—as in the current study—could present cognitive impairment when tested in more complex MWM problems, such as reducing the number of trials by block of trials or increasing the interval of time between trials. Perhaps in more difficult MWM problems, more subtle learning or memory deficits would appear. Another possibility for the absence of effects in this study is that impairments are more evident when prenatal IDA is paired with other nutrient deficiencies. For example, Baumgartner et al. reported several studies in which the effects of prenatal ID were enhanced when coupled with a n-3 fatty acid deficiency [25, 26]. It is possible that the impact of prenatal IDA on learning and memory processes is only visible when paired with other nutrient deficiencies. Despite all these possibilities, the most logical conclusion for the lack of effect in the MWM is that moderate prenatal IDA, as induced by the diet used in the current study, does not influence the progeny’s memory or learning processes.

## Conclusion

The current study emphasizes the need for replication, as recently highlighted by several authors, such as Yong [75], Russell [76], Cesario [77] and Simons [78]. The results of our previous study were controversial with the literature, stating that prenatal IDA produces an increase in exploratory behaviour in guinea pig offspring instead of the much-observed decrease [32, 36, 38, 44] and that there were no visible effects on learning or memory, whereas many had seen deficits [23, 38–40]. However, the current study, by using more precise tools (video tracking systems and behavioural analysis softwares) and analyzing additional variables, supports and refines our previous conclusion. In addition to elevated activity levels in the OFTs (although solely on PNd40) and no effects within the MWM, our pups from the IDA group presented particularly low survival rates, as in Fu et al. [30]. The practice of replicating the previous study has also brought forth several hypotheses as to the cause of this increase in activity, mainly that prenatal IDA provoked augmented anxiety, and that these effects may emerge later in life. As for the lack of effect on learning and memory processes, one could argue that the IDA diet was relatively moderate and that a highly IDA diet could result in significant effects. However, given the high mortality rate associated with the IDA diet in this study, we do not ethically support this idea. In addition, our research was aimed at mimicking the IDA diet consumed by pregnant North American women, which is known for being moderately IDA [79], and it would be inappropriate to develop an animal model based on a highly IDA diet.

## Supporting Information

S1 Results Dam and pup food intake. (PDF)
