# Supplementary material for: Prenatal Iron Deficiency in Guinea Pigs Increases Locomotor Activity but Does Not Influence Learning and Memory
Source: PLoS One. 2015 Jul 17;10(7):e0133168. doi: 10.1371/journal.pone.0133168 (PMC4506089; doi:10.1371/journal.pone.0133168)
Supplement: S1 Results — (PDF) [file pone.0133168.s001.pdf]

Supporting information

Results_S1.pdf

Dam and Pup Food Intake

Dam and pup food intake was measured daily. However, given that both dams and pups were caged in groups of two to six, individual food intake was estimated by dividing the total amount of food eaten by the number of animals within a cage. Consequently, because several animals were attributed the same amount of daily food intake, one should be very careful before drawing any serious conclusion from these data. Nevertheless, given that individual food intake is an important variable when investigating the impact of nutrition, we are providing these data in the current supporting information section.

Dam Food Intake

Due to the fact that the dams were caged in groups of two to six for the duration of the study (see Method), it was not possible to acquire individual food intake data. Nevertheless, the total amount of food eaten (in grams) per cage was divided by the number of animals within the cage to obtain an estimate of individual food intakes. Given that food intakes varied largely from day to day, when observing the amount of food eaten during a particular period, food intakes during that week were averaged.

During the habituation phase, given that the phase was shorter than seven days, solely the last three days of this phase were averaged to account for the dams' food intake. Since all animals within a cage were attributed the same daily food intake, the dams' individual food

intakes during this phase could not be statistically compared because the standard deviations within each group would have been underestimated. Therefore, for the habituation phase, instead of comparing individual food intakes, average food intakes for each cage from both groups (ID = 3, IS = 4) were compared by means of a non-parametric Mann-Whitney test. Dams from both groups ate the same amount of food, $U = 1$, $p = 0.114$, suggesting that both groups adapted equally to their new food pellets.

In contrast to the habituation phase, where food intakes for each cage were compared, individual food intakes for the first and last week of gestation were compared for the gestation phase. The beginning of pregnancy varied between dams, so their individual food intakes during the periods of gestation were different. As expected, weekly food intakes increased from the first week of gestation to the last week of gestation, $F(1, 16) = 53.51$, $p < 0.001$. An ANOVA group (ID, IS) x week of gestation (first, last) with repeated measures on the last factor revealed that the IS group dams ate more food than the ID group dams, $F(1, 16) = 5.648$, $p = 0.030$, but there was no interaction, $F(1, 16) < 1$, $p = 0.616$. A possible explanation for this effect could have been that the ID group females became pregnant at an older age that those in the IS group. However, an unpaired t-test revealed that the mean age of females at the beginning of pregnancy was the same for both groups (ID: $M = 102.50$ days, IS: $M = 108.80$ days), $t(16) = 0.984$, $p = 0.340$, 95% CI of the mean difference = -7.27, 19.87. This alternative hypothesis was therefore ruled out.

Pup Food Intake

The pups' food intake was estimated from PNd10 to PNd40, when they were grouped with other pups after weaning. As with the females, their food intakes were estimated by dividing the total amount of food eaten per cage per day by the number of pups in the cage. Given that pups were caged in groups between two and five and that all pups within the same cage had the same estimated food intake, the pups' individual food intake could not be statistically compared because the standard deviations within each group would have been underestimated. Consequently, average food intakes per cage for both groups of pups were compared (the number of cages for each group were: ID = 7; IS = 8) for the period after separation from the dam (PNd10), at the beginning of the testing phase (PNd24) and at the end of the study (PNd40). Moreover, as with the dams, food intake varied daily, so, to get a better estimate of food intake, the amount of food eaten for seven consecutive days for each time period was averaged (PNd10 = mean from PNd10 to PNd16; PNd24 = mean from PNd21 to PNd27; PNd40 = mean from PNd34 to PNd40).

An ANOVA group (IDA, IS) x period (PNd10, PNd24, PNd40) with repeated measures on the last factor did not reveal any significant main effect of group, $F(1, 13) < 1$, $p = 0.379$. However, there was a significant main effect of period, $F(2, 26) = 30.72$, $p < 0.001$. A series of Tukey *a posteriori* tests revealed that the pups' food intake increased from PNd10 to PNd24, but remained stable between PNd24 and PNd40. Finally, and most importantly, there was a significant group x day interaction , $F(2, 26) = 12.79$, $p < 0.001$. A series of paired t-tests showed that, on PNd40 only, pups born from ID dams ate more food pellets than those born from IS mothers.
